# Silk-Soy Alloy Materials: Influence of Silk Types (Mori, Thai, Muga, Tussah, and Eri) on the Structure, Properties, and Functionality of Insect–Plant Protein Blends (II)

**DOI:** 10.3390/ijms26104563

**Published:** 2025-05-09

**Authors:** Nagireddy Poluri, Christopher R. Gough, Joseph Perrotta, Joseph Pinto, Maxwell Cohen, Steven Sanderlin, Christopher Velardo, Anthony Barca, Xiao Hu

**Affiliations:** 1 Department of Physics and Astronomy, Rowan University, Glassboro, NJ 08028, USA; poluri34@students.rowan.edu (N.P.);; 2 Department of Chemistry and Biochemistry, Rowan University, Glassboro, NJ 08028, USA; 3 Department of Biological and Biomedical Sciences, Rowan University, Glassboro, NJ 08028, USA

**Keywords:** silk fibroin, soy protein, composite film, secondary structure, biopolymer, protein–protein interaction

## Abstract

Natural proteins present a sustainable and biocompatible alternative to conventional fossil fuel-derived plastics, with versatile applications in fields ranging from medicine to food packaging. Extending our previous research on silk–corn zein composites, this study utilizes soy protein—another plant protein extensively employed within biomedical applications—in conjunction with silk fibroin proteins extracted from a variety of domestic (Mori and Thai) and wild (Muga, Tussah, and Eri) silkworm species. By combining these proteins in varying ratios (0%, 10%, 25%, 50%, 75%, 90%, and 100%), silk–soy films were successfully fabricated with high miscibility. The structural and thermal stability of these films was confirmed through various characterization techniques, including Fourier transform infrared spectroscopy (FTIR), differential scanning calorimetry (DSC), and scanning electron microscopy (SEM). Structural refinements were then achieved through post-water annealing treatments. After annealing, it was observed that when soy protein was introduced into both types of silk, the silks exhibited a greater amount of intermolecular and intramolecular β-sheet content. This phenomenon can be attributed to soy’s intrinsic ability to self-assemble into β-sheets through electrostatic and hydrophobic interactions, which also improved the overall thermal stability and morphology of the composite films. The unique self-assembling properties of soy and its ability to promote β-sheet formation facilitate the customization of the silk source and the soy-to-silk ratio. This adaptability establishes protein-based thin films as a versatile and sustainable option for diverse applications in fields such as medicine, tissue engineering, food packaging, and beyond.

## 1. Introduction

Biomaterials play a crucial role in bioengineering and are essential in fields such as medicine, materials science, pharmaceuticals, and environmental science. Protein polymers, historically valuable for their use in textiles and as food ingredients, are naturally highly compatible with the human body, making them ideal for biomedical devices. Several natural proteins such as silk, soy, corn zein, collagen, keratin, and elastin are widely used in various applications due to their unique properties [1]. These natural biomaterials are particularly valuable due to their molecular structures. β-sheets, random-coils, and triple-helices are commonly observed in these proteins, arising from the specific arrangement of their amino acids. The long-range ordering of these amino acids plays a crucial role, as it enables a diverse range of physical, chemical, electrical, and optical properties, all determined by the precise sequence and organization of the amino acids [2].

Silk proteins are fibrous materials primarily derived from spiders and silkworms. For thousands of years, silk from these insects has been used to produce clothing and various furnishing items. Natural silk fibers feature a crystalline, antiparallel β-sheet configuration, which imparts exceptional tensile strength, low weight, and overall toughness to this polymer [2]. Silk materials can be regenerated with tunable proportions of crystalline and amorphous structures, enabling diverse applications. In our study, we investigated two categories of silk: wild silks (*Muga, Tussah*, and *Eri*) and domestic silks (*Mori* and *Thai*). Mori silk, derived from Chinese *Bombyx mori* (*B. mori*) silkworms, is typically white and widely used due to its consistent quality and properties. Thai silk, on the other hand, is a naturally occurring biopolymer extracted from the cocoons of *B. mori* silkworms native to Thailand. Environmental differences influence the Thai silkworm, resulting in yellow cocoons that are slightly smaller than those of the Mori silkworm. Wild silks often exhibit greater mechanical stiffness compared to their domesticated counterparts. Tussah silk, characterized by its dark tan color, is produced by *Antheraea mylitta*. Muga silk, light tan in color, is harvested from *Antheraea assamensis*, while the white Eri silk is obtained from *Philosamia ricini*. These variations in color, origin, and mechanical properties highlight the diversity and unique characteristics of these silk materials, which are shaped by both species and environmental factors.

Silk has been found to exhibit three distinct polymorphs [3,4,5]. Prior to crystallization, silk in an aqueous solution adopts a granular Silk I structure, which can be transformed into a β-sheet-dominated Silk II through heat treatment or physical spinning. Alternatively, Silk I can be converted into a Silk II structure when exposed to alcohol or potassium chloride (KCl) [6] or even through water annealing. The β-sheets in silk organize themselves in an asymmetric structure, with methyl side chains positioned opposite to hydrogen side chains. As a result, this arrangement allows opposing β-sheets to form interlocking interfaces stabilized by hydrogen bonds and van der Waals forces [3]. Some FTIR studies have confirmed an increase in the presence of β-sheets and a decrease in random coils during the transition from Silk I to Silk II [7]. The final morphological properties of silk materials are also influenced by the choice of solvent used for dissolving silk. Studies with various solvents have shown that silk typically has a molecular weight ranging from 250 to 450 kDa after dissolution [8]. In addition, silk can adopt a third structure, known as Silk III, when it forms at an air/water interface [3].

Soybean products are widely consumed worldwide and are highly versatile in their forms of use. Soy proteins are incorporated into diets in various ways, including whole soybeans, soymilk, and soy protein isolates. On a molecular level, soy proteins primarily consist of two small globular proteins: conglycinin (7S) and glycinin (11S). These proteins are predominantly coil-dominated structures, each comprising subunits with varying molecular weights. While most soy proteins are globular and dissolve in salt solutions, a smaller fraction belong to the water-soluble albumin class [9]. Soy proteins typically denature at temperatures ranging from 64 °C to 80 °C [10]. Despite their relatively low denaturation temperature, soy proteins demonstrate significant success in biomedical applications when processed into films [11,12]. In addition, soy protein isolates contain functional groups that can interact with the environment and other molecules, making them useful as functional materials in applications such as filtration [13] and oxidation [14].

By combining natural proteins, such as silk, with plant-derived proteins like soy, protein composites can be tailored for specific applications. This approach builds on our previous work [15], where we explored the interaction of insect and plant proteins through silk–corn zein composites. Materials fabricated from these hybrid polymers are particularly valuable in fields such as medical research, as they can easily interact through charge–charge interactions or hydrogen bonding within the protein structure. This adaptability facilitates their use in addressing a wide range of challenges [2,16,17,18,19,20,21]. Blending is the process of mixing proteins in varying ratios to fabricate materials with properties that can be tuned for specific applications [1]. The material processed by blending may possess various mechanical [22,23], electrical [24], chemical [25], biological [26,27], or optical qualities [28]. The desired material properties can be achieved by fine-tuning the ratios used in the blending process. Additionally, the variations in the molecular structure of the blends allow for tunable characteristics, such as elasticity [29], biodegradability, and biocompatibility [30] in various forms including gels [31], films [19,32,33,34,35,36], and fibers [37,38,39,40,41]. Blending protein polymers, as opposed to synthetically engineering copolymers, is often more cost-effective and efficient than meticulously creating a polymer through chemical or biological reactions. This approach allows the biomaterial to be more easily crafted for a specific purpose. In this study, thin films were fabricated using varying ratios of silk and soy proteins, with a range of silks included to explore how these mixtures influence the material properties. The effect of deionized water as a coagulation agent was also investigated. To assess the miscibility of the proteins and quantify the resulting material properties, a series of morphological, structural, and thermal tests were performed on the samples.

## 2. Results and Discussion

### 2.1. Structural Analysis

**Figure 1:**
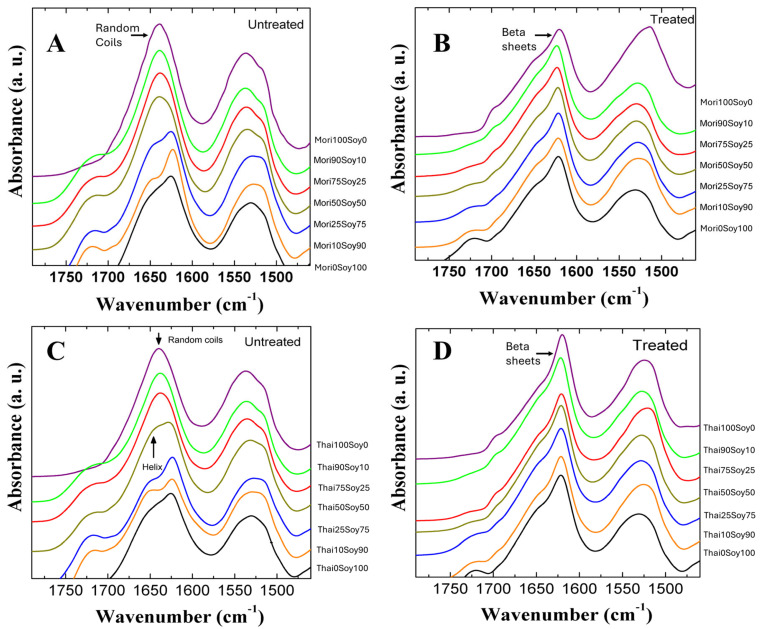
FTIR spectra of Mori (**A**,**B**) and Thai (**C**,**D**) silk–soy films before (**A**,**C**) and after (**B**,**D**) water annealing treatment.

**Figure 2:**
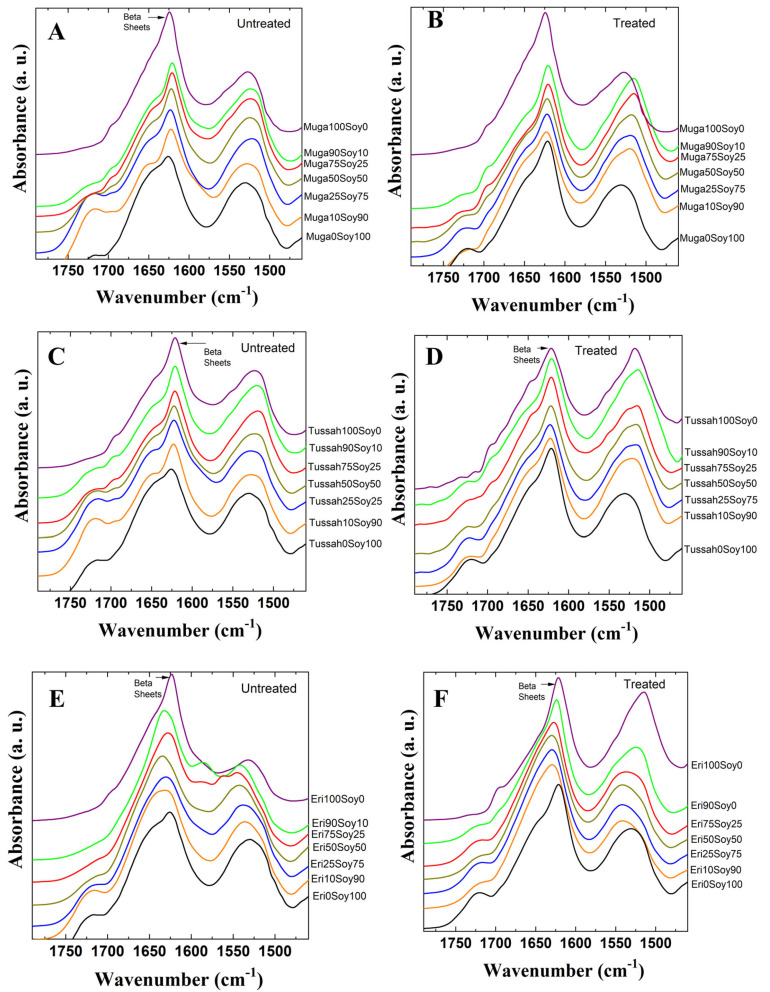
FTIR spectra of Muga (**A**,**B**), Tussah (**C**,**D**), and Eri (**E**,**F**) silk–soy films before (**A**,**C**,**E**) and after (**B**,**D**,**F**) water annealing treatment.

FTIR spectroscopy was utilized to examine the protein secondary structure composition of silk–soy composite films. Samples were analyzed both before and after annealing in water to evaluate changes in structure. The analysis included domestically sourced silk (Mori and Thai, Figure 1) and wild-sourced silk (Muga, Tussah, Eri, Figure 2), focusing on structural differences between silk types, their interactions with soy protein at varying ratios, and the effects of water annealing treatment on their structures.

For untreated samples, the pure silk (100%) exhibited a random coil structure, as indicated by the peak at 1640 cm^−1^ in Figure 1. Introducing soy into the composite led to an increase in β-sheet content proportional to the soy concentration, while random coil content showed a corresponding decrease. For example, incorporating soy into untreated Mori silk proteins exhibited a significant proportion of intramolecular β-sheets especially in Mori25-Soy75 and Mori10-Soy90 samples as evidenced by absorbance peaks in the Amide I region around 1620 cm^−1^ (Figure 1A). This phenomenon can be attributed to soy’s intrinsic ability to self-assemble into β-sheets through electrostatic and hydrophobic interactions [42]. Overall, untreated silk-dominant films exhibit a predominance of random coil structures, whereas soy-dominant blends are characterized by a higher β-sheet content. Similar trends are evident in water-annealed samples, as shown in Figure 1B; however, the β-sheet content in all silk-containing samples is notably higher after water treatment, which can be indicated by the shift in dominated peak position in Amide I from 1640 to 1620 cm^−1^. This increase can be attributed to the removal of residual Ca²⁺ ions introduced by the solvent. These ions hinder β-sheet crystalline formation by promoting ionic interactions, and their removal, therefore, facilitates the stabilization of β-sheet structures [43].

The Amide I and II absorption bands for Thai–soy blended films are presented in Figure 1C,D. Among all the composites tested, Thai-dominant samples primarily exhibit random coil structures similar to Mori-dominant samples. However, in soy-dominant Thai and Mori samples, a distinct peak at 1650 cm^−1^ associated with α-helix (and/or β-turns) structures emerges with the clear trend observed in the FTIR spectra. Water annealing significantly enhances the structural stability of all Thai silk composite films by promoting β-sheet crystalline formation across all samples. This structural transition is evident in the FTIR spectra, where the peaks corresponding to intermolecular β-sheet structures become more pronounced at 1620 cm^−1^ observed in Figure 1D like Mori samples [44]. After water annealing, a small peak around 1695 cm⁻¹ was also observed in the domestic silk–soy composites, which is indicative of antiparallel β-sheet structures [45]. The results of both domestic silk samples suggest that water annealing facilitates the reorganization of protein secondary structures, increasing β-sheet crystalline content while influencing the overall conformational balance making the composites more suitable for biomedical and industrial applications with a strong long-range β-sheet crystalline structure.

Figure 2 presents the Amide I (1600–1800 cm^−1^) and Amide II (1470–1570 cm^−1^) spectral regions for Muga–soy (A,B), Tussah–soy (C,D), and Eri–soy (E,F) composite films, analyzed at different silk-to-soy ratios. The left column shows untreated samples, while the right column displays those subjected to water annealing. The data reveal that pure soy primarily adopts an intramolecular β-sheet structure, as indicated by a peak near 1626 cm^−1^. In contrast, pure wild silk films exhibit a combination of intra- and intermolecular β-sheet structures, with a characteristic peak around 1622 cm^−1^. As the proportion of wild silk increases in the composite films, the secondary structure shifts, leading to a slight change in the β-sheet peak position.

The impact of water annealing treatment on these films is evident in the right column of Figure 2. After annealing, soy-rich films tend to adopt more β-sheet structures (with a decrease in α-helix peaks) and shift the peak position to 1620 cm^−1^, whereas silk-rich composites slightly strengthen their intermolecular β-sheet networks at a similar wavenumber. This transformation occurs because water annealing facilitates molecular rearrangement by enhancing hydrogen bonding while simultaneously reducing interference from external ions. As a result, the β-sheet structures become more stable and well-ordered. Consequently, this structural reorganization improves both the stability and thermal integrity of the silk–soy composites. Therefore, these results from FTIR highlight the crucial role of intermolecular β-sheets in determining the physical performance of these hybrid films. More importantly, they demonstrate how the material properties can be systematically fine-tuned through variations in composition and processing techniques, offering potential for targeted applications in biomaterials and advanced composites.

### 2.2. Thermal Analysis

**Figure 3:**
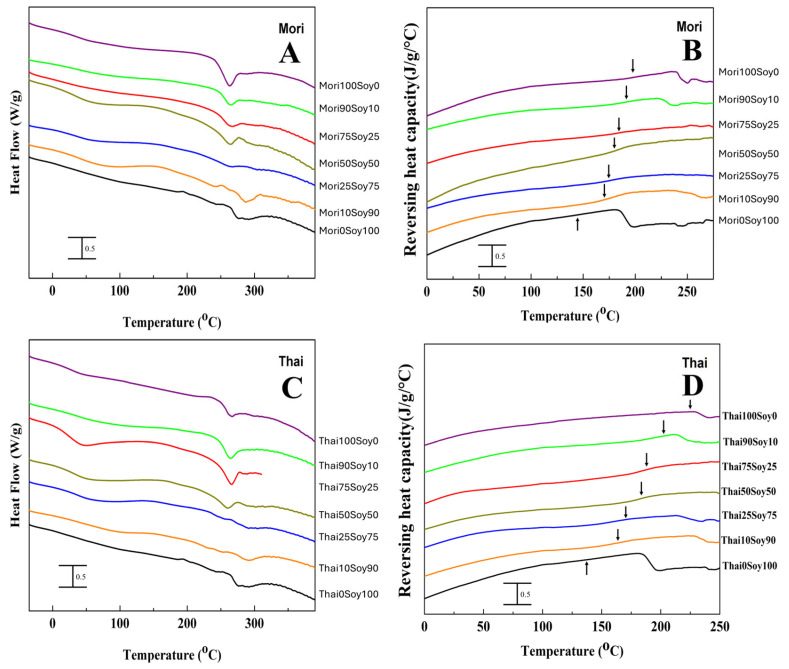
(**A**,**C**) Total heat flow and (**B**,**D**) reversing heat capacity of (**A**,**B**) Mori–soy and (**C**,**D**) Thai–soy composite films (glass transition temperatures are indicated by arrows in (**B**,**D**) for domestic silk–soy composites).

**Figure 4:**
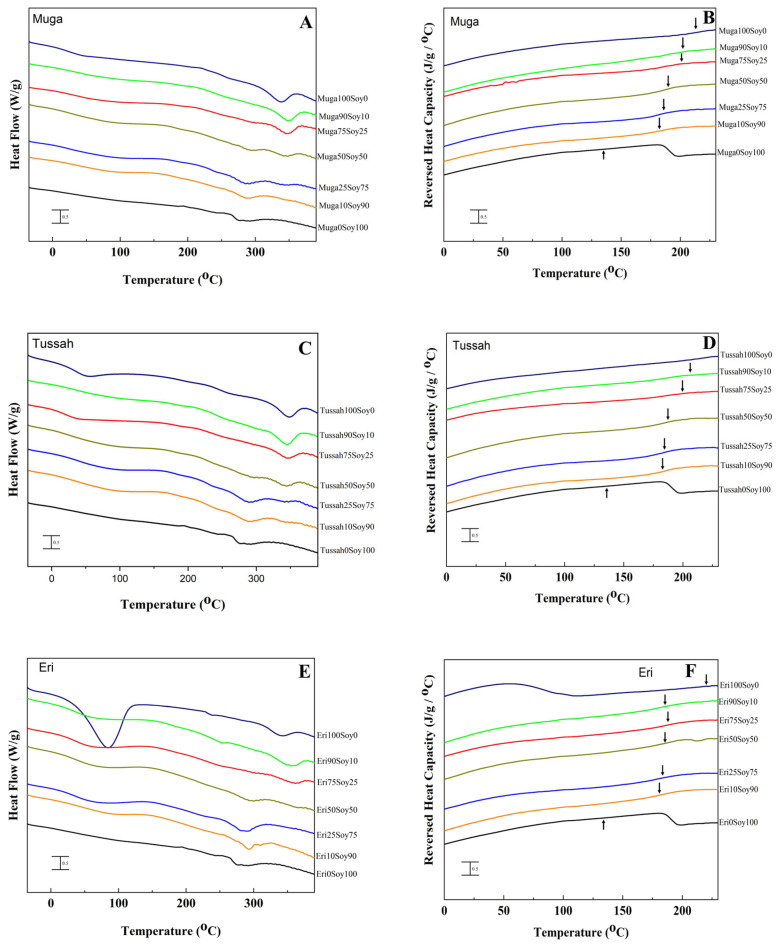
Total heat flow (**A**,**C**,**E**) and reversing heat capacity (**B**,**D**,**F**) from DSC analysis of (**A**,**B**) Muga–soy, (**C**,**D**) Tussah–soy, (**E**,**F**) and Eri–soy composite films (glass transition temperatures are indicated by arrows in (**B**,**D**,**F**) for wild silk–soy composites).

Several findings from the DSC thermal analysis support the observations made in the FTIR analysis. The glass transition and degradation temperatures of various silk–soy films are depicted in Table 1 for both domestic silk and wild silk composites. As noted earlier, ordered protein conformations exhibit greater thermal stability than less ordered structures, which is reflected in the thermal degradation process. Composite films made from domestic silks (Figure 3, Mori, Thai) degrade at significantly lower temperatures than their wild counterparts (Figure 4, Muga, Tussah, Eri). Specifically, the degradation temperatures (*T*_d_) of Mori–soy composite samples range from 262.7 °C to 287.1 °C, while those of Thai–soy composites range from 264.8 °C to 292.9 °C (Table 1). In contrast, wild silk composite films exhibit higher thermal stability, with Muga–soy composites degrading between 338.9 °C and 286.4 °C, Tussah–soy composites between 348 °C and 288.3 °C, and Eri–soy composites between 344.6 °C and 292.9 °C (Table 1). A similar trend is seen in the glass transition temperatures (*T*_g_), which mark the transition from a rubbery state to a hard, glassy state. Domestic silk films (Mori and Thai) exhibit a *T*_g_ at temperatures of 179.1 °C and 218.2 °C, respectively, while wild silk–soy blends (Muga, Tussah, Eri) show *T*_g_ at 212.8 °C, 231.7 °C, and 220.1 °C. Despite the differences in *T*_g_, both domestic and wild silk demonstrate good miscibility with soy protein, as indicated by the absence of multiple glass transitions in a single composite material.

Beginning with Mori–soy films in Figure 3A,B and in Table 1, no significant differences in degradation or glass transition temperatures are found with changing composition ratios. However, samples with higher amounts of soy exhibit slightly higher degradation temperatures and lower glass transition temperatures than those with lower soy content. These films with high soy content degrade at temperatures ranging from 266.9 °C to 287.1 °C, while films with lower soy content degrade around 262.7 °C to 265.1 °C. The 25% and 50% Mori samples show two degradation peaks, with the second peak occurring much higher than the first, at 302.8 °C and 298.6 °C, respectively. In contrast, higher soy content results in a lower glass transition temperature. Regardless, the distinct peaks in degradation, and especially the lack of separate degradation peaks for most composites, show that Mori silk and soy were able to form stable composites.

Thai–soy composite films show similar properties to Mori–soy films, mirroring their similarities in FTIR analysis. The glass transition temperature shows a decrease in *T*_g_ with an increase in soy, falling from 198.1 °C at 90% Thai down to 167.2 °C at 10% Thai (Table 1). Thai–soy composite films containing less than 25% silk have weak degradation peaks (Figure 3C), where increasing soy content generally increases the degradation temperature (Table 1). At higher concentrations of silk, however, the clear glass transition temperatures and strong single degradation peaks indicate that the two proteins form stable composites.

Silk–soy blends made with silk from wild silkworm sources (Figure 4) exhibit higher thermal stability due to their intramolecular β-sheet structural composition, as observed from FTIR. Regarding the ratio of silk to soy in each sample set, many of the trends observed in domestic silk are mirrored. Specifically, in Muga–soy composites, Muga films without soy show a glass transition temperature of 212.8 °C, which decreases with an increase in soy content in the blend film (Table 1). The pure Muga films also exhibit a high single degradation peak at 338.9 °C due to their stable molecular structure. However, pure Muga does not exhibit the highest degradation temperature; the most thermally stable Muga–soy composition appears to be the 50% blend, which has two degradation peaks, with the higher one related to the Muga silk component at 344.8 °C. This indicates that Muga silk proteins have a strong interaction with the soy proteins and enhance the structural stability of the blends during mixing. The lower degradation peak for this sample is at 294 °C, which is also higher than that of the pure soy protein film at about 279.3 °C (Table 1). Beyond this point, degradation temperatures decrease as more soy is added, indicating reduced thermal stability. In addition, the glass transition temperature occurs at lower temperatures and with a more pronounced shift as soy content increases.

Tussah silk exhibits structural differences compared to Muga silk, as indicated by the FTIR analysis. However, this variation does not significantly impact the thermal properties of the films when compared to Muga–soy films. Similar to Muga–soy composites, films made with Tussah silk maintain strong thermal stability, with silk concentrations ranging from 10% to 90% supporting high degradation temperatures, between 288.3 °C and 345.4 °C (Table 1). Despite this, 100% Tussah films exhibit the highest degradation temperature at 348 °C, with a glass transition temperature of 231.7 °C; both are the highest observed in all silk–soy composites. As more soy is incorporated, the glass transition temperature of Tussah silk–soy films decreases, eventually reaching 178.4 °C for 10% Tussah silk films. A similar trend is observed in the initial degradation temperature, which declines accordingly.

The glass transition temperature trend of Eri silk–soy films is similar to those observed in other silk–soy composite films. Eri silk degrades at a temperature of 344.6 °C, but the highest degradation temperatures are found in composites with Eri silk of 90% and 75% (silk-dominant samples), where *T*_d1_ reaches 308.2 °C and *T*_d2_ reaches 358.8 °C for the 75% Eri composite films. Interestingly, samples with less Eri silk content (25% and 10%) do not show a clear second degradation peak, *T*_d2_, in the DSC curves, indicating that the interaction of Eri silk with the soy proteins could be more complicated compared to Muga and Tussah silks. This finding also echoes the FTIR results that Eri silk–soy protein films tend to have weaker β-sheet peaks in the Amide I region (Figure 2E,F) compared to the spectra from Muga–soy and Tussah–soy samples (Figure 2A–D) with the same mixing ratios.

### 2.3. Morphology Analysis

**Figure 5:**
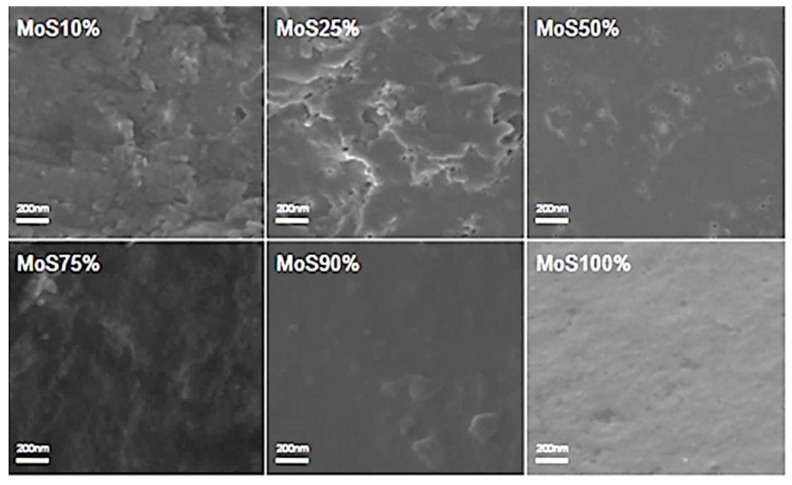
SEM images illustrating the microstructure of Mori–soy protein composite films with silk fibroin content ranging from 10% to 100% (scale bar: 200 nm).

**Figure 6:**
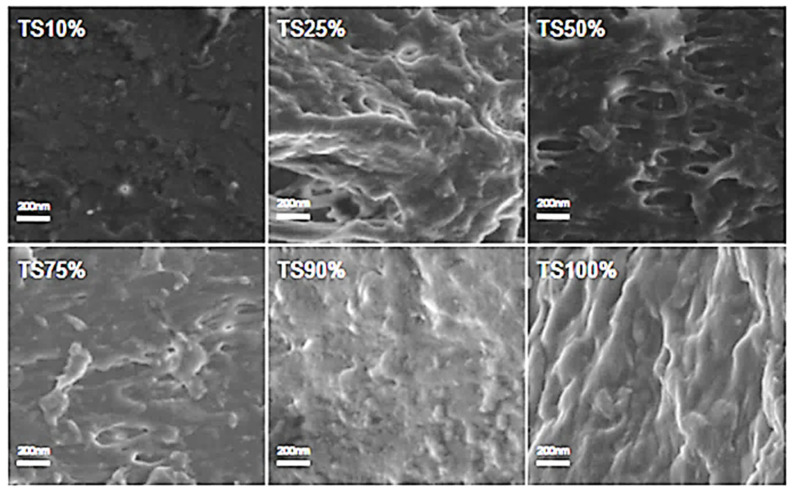
SEM images illustrating the microstructure of Thai–soy protein composite films with silk fibroin content ranging from 10% to 100% (scale bar: 200 nm).

**Figure 7:**
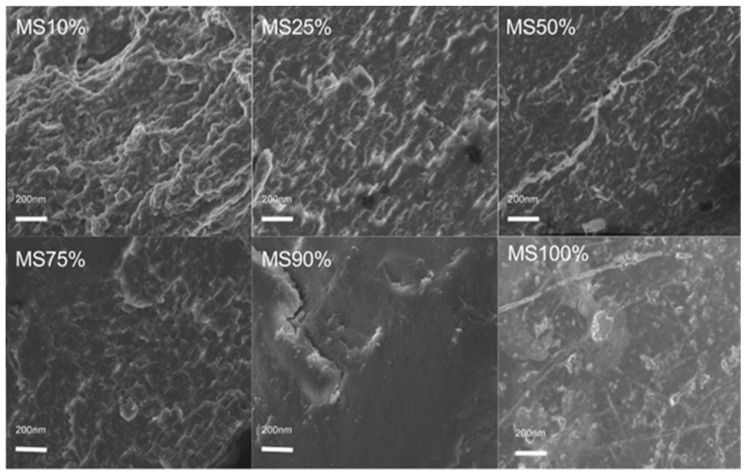
SEM images illustrating the microstructure of Muga–soy protein composite films with silk fibroin content ranging from 10% to 100% (scale bar: 200 nm).

**Figure 8:**
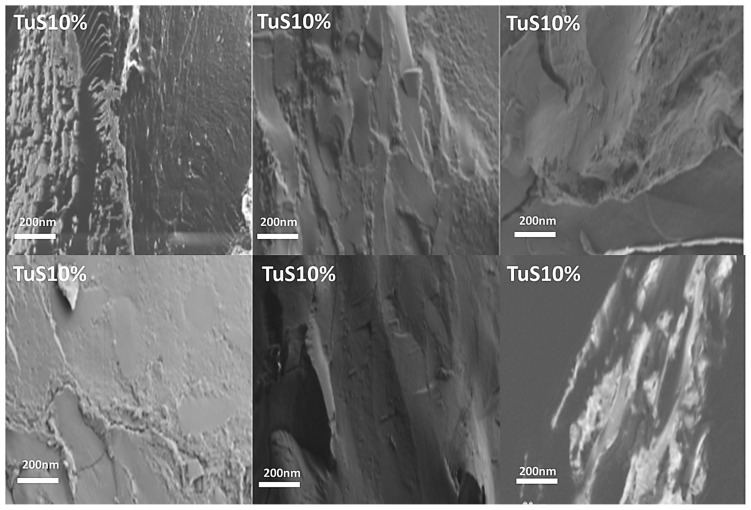
SEM images illustrating the microstructure of Tussah–soy protein composite films with silk fibroin content ranging from 10% to 100% (scale bar: 200 nm).

**Figure 9:**
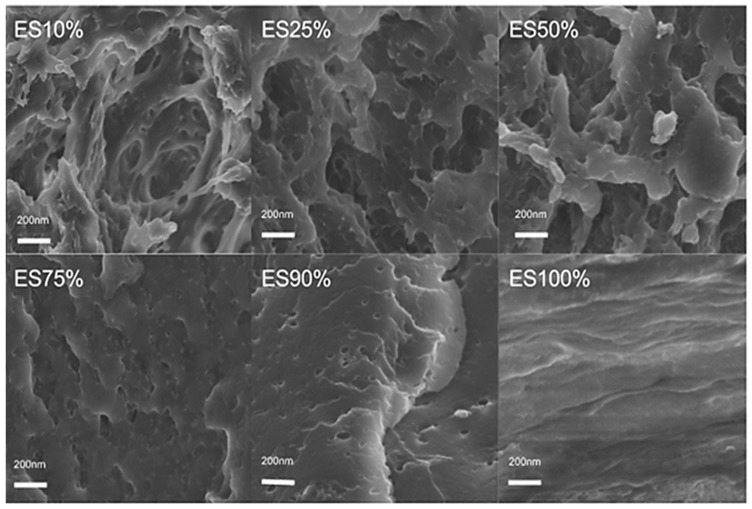
SEM images illustrating the microstructure of Eri–soy protein composite films with silk fibroin content ranging from 10% to 100% (scale bar: 200 nm).

**Figure 10:**
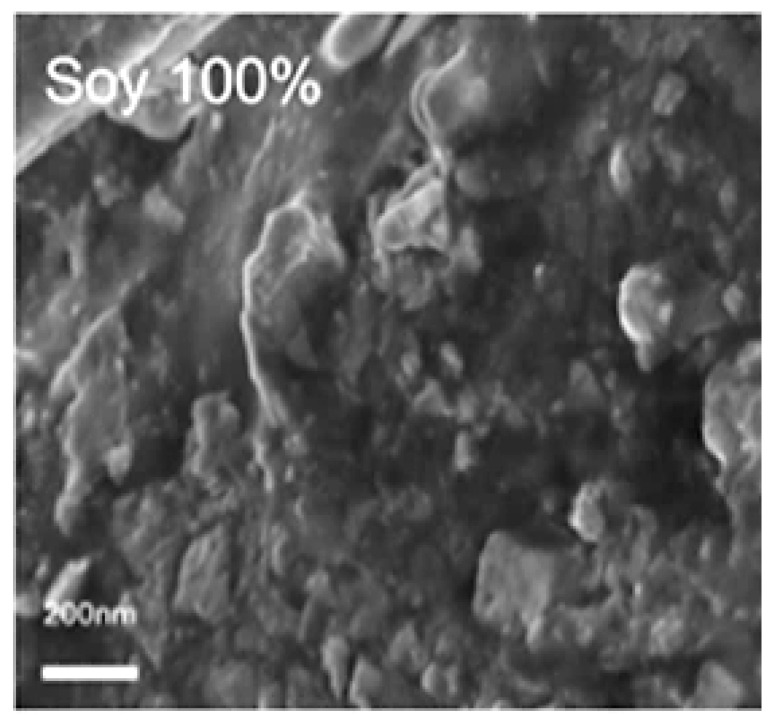
SEM images showing the microstructure of pure soy protein films. Images were captured at a scale bar of 200 nm.

Scanning electron microscopy (SEM) was used to analyze the cross-sectional microstructure of untreated silk–soy films at a 200 nm scale. Figure 5 and Figure 6 illustrate the microstructural differences across films made from domestic silks (Mori, Thai) and soy protein, with silk content ranging from 10% to 100%. Figure 7, Figure 8 and Figure 9 display data for wild silk (Tussah, Muga, and Eri) composites. Figure 10 shows the pure soy protein film as a comparison. The microstructural progression in Mori silks, as the silk percentage increases, demonstrates a decrease in surface roughness. As soy protein becomes more prominent in the films (e.g., MoS25%, MoS10%), it introduces greater rigidity due to the presence of intramolecular β-sheets in soy proteins. At 100% Mori silk (MoS100%), a smooth microstructure is observed, inherent to the natural fibrillar structure of Bombyx mori silk [46]. Thai silk–soy films (TS10–TS100%) exhibit a different micromorphology, with a wave-shaped pattern running longitudinally along the cross-section. This longitudinal growth is also characteristic of the properties found naturally in Mori silk fibroin films [44,46,47].

Wild silk films exhibit a very similar microstructural progression to domestic films, with surface roughness decreasing as the silk percentage increases (Figure 7, Figure 8 and Figure 9). Pure wild silk films (MS100%, TuS100%, ES100%) are generally smooth at a 200 nm scale compared to their soy composite samples, while Muga silk also shows many tiny ‘blobs’ along the smooth surface. Eri–soy films with high soy percentages all show ridge-like features, which are due to the complicated interaction of Eri silk protein with the soy protein, as we found in the thermal and spectral studies. Particle-like shapes are clearly seen in Figure 10, which is an SEM image of a pure soy protein film [47], since they are typical globular proteins and tend to self-assemble into particles at the nanoscale. The soy protein matrix in the composites is disrupted by the addition of more intermolecular silk β-sheet structures in the films, causing the particles to open up and form porous scaffold shapes in the cross-section. These variations in structural topography and component orientation highlight the impact of silk type on the composite’s nanostructure, indicating better miscibility in our protein blend films which was also evident from the single glass transition observed in the DSC study. Therefore, by systematically tuning the silk-to-soy ratio and modifying the physical and chemical characteristics of the films, we can provide flexible biomaterial composites that are suited to specific application needs in biomedical science.

### 2.4. Mechanism

**Figure 11:**
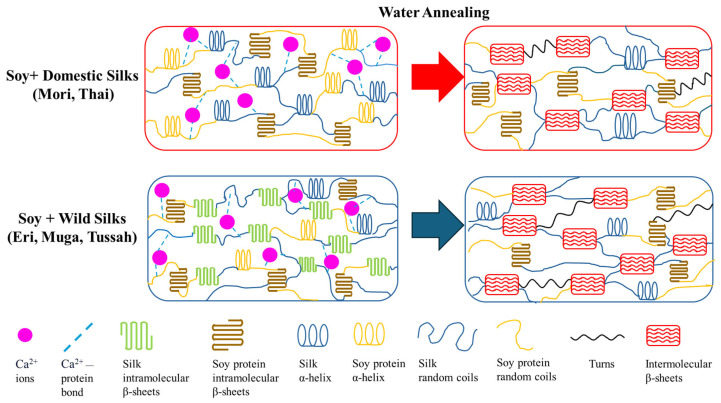
Schematic illustration of the protein structure in silk–soy composite films before and after water annealing treatment.

The structural changes in silk–soy composite films can be explained by the mechanism illustrated in Figure 11. The structural properties of these films exhibit significant dependence on their composition ratios and post-treatment conditions, as revealed through FTIR and thermal analysis. In untreated domestic silk films, the presence of calcium ions (Ca²⁺) disrupts hydrogen bonding, inhibiting the formation of stable intermolecular β-sheets. After treatment with water annealing, these ions are removed, and new hydrogen bonds are formed between silk protein molecular chains, allowing silk proteins to transition from random coils and α-helices into intermolecular β-sheet crystalline structures. This structural shift is evident from the FTIR Amide I peak moving from 1640 cm^−1^ to 1620 cm^−1^. Similarly, wild silk films experience a reorganization of intramolecular β-sheets into more stable long-range intermolecular networks, emphasizing the critical role of water annealing treatment in enhancing protein conformation.

Introducing soy protein into the composite matrix further influences its structural profile. With increasing soy content, there is a proportional rise in β-sheet formation, accompanied by a reduction in random coil structures, as observed with the shift in FTIR peaks. This behavior can be attributed to soy’s inherent ability to self-assemble through electrostatic and hydrophobic interactions. Thermal analysis using DSC highlights a corresponding decline in degradation temperature and glass transition temperature with higher soy concentrations, reflecting increased flexibility due to soy interactions with silk.

In post-treatment films, the structural enhancements are even more pronounced, with silk-dominant compositions retaining more β-sheet crystalline structures. The removal of Ca^2+^ ions not only facilitates stronger hydrogen bonding within the silk network but also promotes interactions between silk’s β-sheets and soy’s flexible helix (and/or β-turns) and coil structures. This interplay leads to a shift in the FTIR Amide I peak, indicating backbone alterations, and a sharpening of the Amide II peak, highlighting enhanced side-chain mobility.

## 3. Materials and Methods

### 3.1. Preparation of Materials

**Figure 12:**
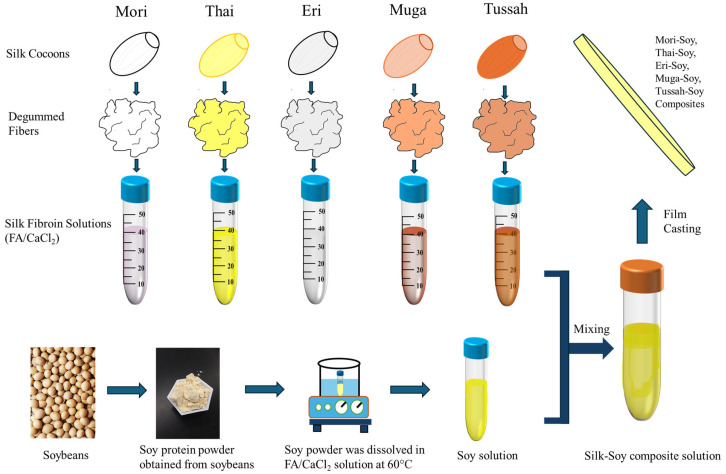
Fabrication procedure of various silk–soy protein composite films.

Five different types of silkworm cocoons used in this study were obtained from various sources (Figure 12). Three wild-type silks, *Philosamia Ricini* (Eri), *Antheraea Assamensis* (Muga), and *Antheraea Mylitta* (Tussah), were acquired from India, while two domestic *B*. *mori* silk cocoons were sourced from China (Mori) and Thailand (Thai). The solvent used to dissolve the silk fibroin and soy protein was a 4 wt.% calcium chloride–formic acid (FA/CaCl_2_) solution, and both calcium chloride powders and formic acid (ACS grade, 98% pure) were purchased from EMD Millipore Corporation (Burlington, MA, USA).

All silkworm cocoons were processed as follows: Sericin was removed by boiling the cocoons in a 0.02 M Na_2_CO_3_ aqueous solution while stirring for 2 h. The degummed cocoons were then subjected to three 20 min washes with deionized (DI) water, replacing the water after each wash. Silk fibroin fibers were subsequently dried overnight in a fume hood.

Silk–soy protein films were prepared by first dissolving soy protein powder in 17.5 mL of FA/CaCl_2_ solution in one test tube, heated to 60 °C and continuously stirred to facilitate dissolution, while the silk fibroin was dissolved separately in the FA/CaCl_2_ solution. Once the soy protein was fully dissolved, both silk fibroin and soy protein solutions were combined to produce blends with varying silk-to-soy ratios of 0:100, 10:90, 25:75, 50:50, 75:25, 90:10, and 100:0 by weight percentage. The resulting solutions were poured into polydimethylsiloxane (PDMS) molds and dried for approximately 24~48 h in a fume hood. The final untreated films had an approximate thickness of 25 µm. Additional samples were subsequently annealed in deionized water for 30 min and then dried overnight again in the fume hood.

### 3.2. Fourier Transform Infrared Spectrometry (FTIR)

FTIR measurements were conducted using a Bruker Tensor 27 Fourier Transform Infrared Spectrometer (Billerica, MA, USA), equipped with a deuterated triglycine sulfate detector and a multiple-reflection horizontal MIRacle ATR attachment (with a Ge crystal from Pike Tech, Madison, WI, USA). Spectra were recorded in the range of 4000 cm⁻¹ to 400 cm⁻¹, with a resolution of 4 cm^−1^ and 64 scans per measurement. To ensure consistency across the films, each sample was measured at different regions (n ≥ 4), and the representative spectra was averaged for all samples before and after treatment. The ATR crystal was cleaned with ethanol between each measurement to remove any residues.

### 3.3. Differential Scanning Calorimetry (DSC)

Approximately 6 mg samples of water-treated films were placed in aluminum pans and heated in a TA Instruments Q100 DSC (New Castle, DE, USA), which was purged with a dry nitrogen gas flow (50 mL/min) and equipped with a refrigerated cooling system. Measurements were taken from −40 °C to 400 °C at a heating rate of 2 °C/min, with a modulation period of 60 s and a temperature amplitude of 0.318 °C. Prior to measurements, calibration was performed using an indium standard for heat flow, and the instrument was calibrated for heat capacity using aluminum and sapphire standards. To ensure consistency of the results, the samples were tested in triplicate for each condition.

### 3.4. Scanning Electron Microscopy (SEM)

Cross-sectional images of the water-treated blended films were taken to examine the structural characteristics of silk–zein films at different composition ratios. Imaging was performed using a Phenom Pure scanning electron microscope (Eindhoven, Netherlands) with an electron high tension (EHT) of 10 kV, and magnifications at 200 nm. Cross-sectional SEM samples were prepared by immersing the films in liquid nitrogen, followed by fracturing them into small pieces. The sample stages for cross-sectional imaging were prepared by placing a flat strip of double-sided carbon tape against the surface of the stage, positioning the film at a 90° angle. Microscopic images were acquired from multiple locations across the film’s cross-sectional surface to assess and confirm structural homogeneity. Prior to imaging, all samples were sputter-coated with gold for 15 s using a Denton Vacuum Desk II sputtering machine (Moorestown, NJ, USA) to enhance image resolution.

## 4. Conclusions

FTIR, DSC, and SEM analyses were used to confirm the structural and thermal integrity of thin films made from various silks, soy, and their composites. Annealing with deionized (DI) water introduced additional variations in the material properties of the silk–soy protein composite films. Domestic silk exhibits lower β-sheet content compared to soy protein. Therefore, the incorporation of soy, which naturally self-assembles into β-sheets, enhances the films’ thermal integrity. Water annealing treatment further increases the β-sheet crystallinity in domestic silks and their composites as evidenced in FTIR, enabling fine-tuning of the films’ material properties. In contrast, silk from wild sources naturally contains intramolecular β-sheets before water treatment. Water treatment further strengthens the protein structure, enhancing the thermal stability of the films. Incorporating soy in optimal ratios significantly improves composite structural stability, as confirmed by FTIR and DSC analyses. Based on the findings from this study, it is possible to create biofilms with tailored properties by selecting appropriate silk sources and adjusting silk-to-soy ratios for diverse applications in medicine, packaging, and engineering.

## Figures and Tables

**Table 1:** Glass transition and degradation temperatures of domestic and wild silk–soy composite films. All values are subject to a ±5% error margin.

Sample	*T*_g_ (°C)	*T*_d1_ (°C)	*T*_d2_ (°C)	Reference
Mori100Soy0	179.1	262.7	N/A	[44]
Mori90Soy10	177.8	264.0	N/A	
Mori75Soy25	175.1	265.1	N/A	
Mori50Soy50	174.1	266.9	298.6	
Mori25Soy75	173.8	268.0	302.8	
Mori10Soy90	168.6	287.1	N/A	
Thai100Soy0	218.2	264.8	N/A	[44]
Thai90Soy10	198.1	265.5	N/A	
Thai75Soy25	186.7	265.3	N/A	
Thai50Soy50	186.0	259.4	N/A	
Thai25Soy75	174.7	290.2	N/A	
Thai10Soy90	167.2	292.9	N/A	
Muga100Soy0	212.8	338.9	N/A	[44]
Muga90Soy10	191.2	348.0	N/A	
Muga75Soy25	190.6	346.7	N/A	
Muga50Soy50	185.4	294.0	344.8	
Muga25Soy75	181.2	287.6	342.7	
Muga10Soy90	179.8	286.4	340.8	
Tussah100Soy0	231.7	348.0	N/A	[44]
Tussah90Soy10	201.3	345.4	N/A	
Tussah75Soy25	198.2	343.4	N/A	
Tussah50Soy50	181.5	294.7	341.6	
Tussah25Soy75	179.8	288.9	340.8	
Tussah10Soy90	178.4	288.3	337.6	
Eri100Soy0	220.1	344.6	N/A	[44]
Eri90Soy10	183.7	353.1	N/A	
Eri75Soy25	185.9	308.2	358.8	
Eri50Soy50	181.5	293.9	357.5	
Eri25Soy75	178.0	283.2	N/A	
Eri10Soy90	177.7	292.9	N/A	
Soy100	135.7	279.3	N/A	

## Data Availability

The original contributions presented in this study are included in the article. Further inquiries can be directed to the corresponding author.
